# A Variant of the Sciatic Nerve and its Clinical Implications

**DOI:** 10.7759/cureus.2874

**Published:** 2018-06-25

**Authors:** Graham Dupont, Florence Unno, Joe Iwanaga, Rod J Oskouian, R. Shane Tubbs

**Affiliations:** 1 Neuroscience Institute, Seattle Science Foundation, Seattle , USA; 2 Hansjörg Wyss Hip and Pelvis Center, Swedish Medical Center, Seattle, USA; 3 Seattle Science Foundation, Seattle, USA; 4 Neurosurgery, Swedish Neuroscience Institute, Seattle, USA; 5 Neurosurgery, Seattle Science Foundation, Seattle, USA

**Keywords:** nerve injury, iatrogenic, surgery, anatomy, posterior thigh, gluteal region

## Abstract

Variants of gluteal neural anatomy are important to consider, especially during surgical approaches to the hip. During the routine dissection of the gluteal region, a variant of the sciatic nerve was found where the nerve left the pelvis fully split into its tibial and common fibular components. Intrapelvically and extrapelvically, there was no splitting of the two components by the piriformis muscle. Distally, the two parts of the nerve were draped over the medial and lateral edges of the ischial tuberosity. To avoid iatrogenic injury to the sciatic nerve during invasive or surgical approaches to this region, all possible anatomical variations, such as the one presented herein, should be appreciated by the clinician.

## Introduction

The sciatic nerve is not a singular nerve itself, rather it is a bundle of two nerves—the common fibular and tibial nerves—bound together by a common epineural sheath that approximates them as they course inferiorly down the posterior gluteal region and posterior thigh. The sciatic nerve, usually arising from the L4-S3 segments of the sacral plexus, leaves the pelvis to enter the gluteal region via the greater sciatic foramen and usually courses inferior to the piriformis. The nerve then bifurcates into its two parts at the lower third of the femur near the apex of the popliteal fossa [1]. Variations of the sciatic nerve, especially in the gluteal region, are not uncommon. These documented high divisions include those in which the common fibular nerve pierces or exits superior to the piriformis, and the tibial nerve courses below the piriformis in normal fashion [1]. In this report, we discuss the high division of the sciatic nerve not involving the piriformis.

## Case presentation

**Figure 1:**
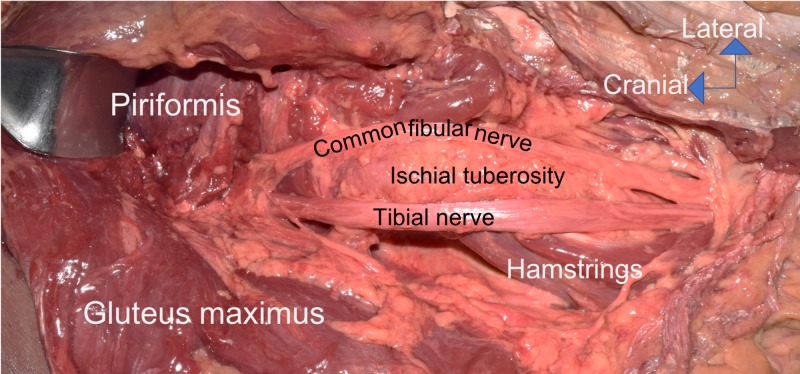
Variant right sciatic nerve. Note the sciatic nerve is united just inferior to the piriformis muscle. However, distal to this location, the nerve is split around the ischial tuberosity with the tibial nerve being located lateral to the ischial tuberosity. Distal to this, the two components travel more closely together but remain ununited.

During the routine dissection of the right gluteal region and upper posterior thigh of an adult male cadaver (aged 74 years at death), the sciatic nerve was found to have an unusual configuration. Specifically, the nerve left the pelvis as a united sciatic nerve, but just inferior to the piriformis it fully split into its tibial and common fibular components (Figure 1). Intrapelvically and extrapelvically, there was no splitting of the two components by the piriformis muscle. Within the pelvis, the tibial and common fibular components of the sciatic nerve were united and derived from L4 to S3 ventral rami. Just inferior to the inferior border of the piriformis muscle, the two parts of the sciatic nerve separated and distally, these were draped over the medial and lateral edges of the ischial tuberosity and therefore, splayed apart (Figure 1). This necessitated the common fibular part of the sciatic nerve to travel over the sacrotuberous ligament and this crossing occurred just inferior to the lowest attachment of the gluteus maximus muscle onto the sacrotuberous ligament (Figure 1). In the posterior thigh, the two parts were more closely approximated but still did not have a common sheath. In the upper aspect of the popliteal fossa, the tibial and common fibular nerves continued in a normal fashion. No other neurovascular or musculoskeletal variants were noted in the areas dissected. Additionally, no signs of pathology or past surgical procedures were noted in this region. Lastly, on the contralateral side, the sciatic nerve was formed in routine fashion and did not split prematurely as found on the right side. Anatomical quality assurance guidelines were followed [2, 3].

## Discussion

Most premature separations of the tibial and common fibular components of the sciatic nerve occur due to the later part piercing or coursing over the piriformis muscle. The case presented herein is unusual in the fact that the sciatic nerve had a normal course as it left the pelvis, but inferior to the piriformis, the nerve separated and was split on either side of the ischial tuberosity. Normally, both parts of the nerve, even when already segregated into parts, travel lateral to the ischial tuberosity. Despite the separation around the ischial tuberosity, the distal splitting of the sciatic nerve just inferior to the piriformis is a type C variant in which the nerve divides at the upper part of the posterior compartment of the thigh (12% occurrence), as demonstrated in the study by Kiros and Woldeyes [4].

Normally, the sciatic nerve, after it leaves the pelvis through the greater sciatic foramen, is located between the greater trochanter (laterally) and the ischial tuberosity (medially) [5,6]. In the case presented herein, if this landmark location were assumed to contain the entire sciatic nerve with injections, electrode placement, acupuncture needle placement, or surgery and the area just lateral to the ischial tuberosity were engaged, then injury to the common fibular nerve might occur (Figure 1) [7,8].

## Conclusions

To avoid iatrogenic injury to the sciatic nerve during invasive or surgical approaches to this region, all possible anatomical variations, such as the one presented herein, should be appreciated by the clinician.
